# Eltrombopag versus romiplostim in treatment of children with persistent or chronic immune thrombocytopenia: a systematic review incorporating an indirect-comparison meta-analysis

**DOI:** 10.1038/s41598-017-19099-8

**Published:** 2018-01-12

**Authors:** Jiaxing Zhang, Yi Liang, Yuan Ai, Xiaosi Li, Juan Xie, Youping Li, Wenyi Zheng, Rui He

**Affiliations:** 1 Chinese Evidence-based Medicine Center, Sichuan University, Chengdu, China; 2 Department of Pharmacy, Guizhou Provincial People’s Hospital, Guiyang, China; 3 Health Outcomes and Pharmacy Practice, College of Pharmacy, the University of Texas at Austin, Texas, United States; 4 Department of Pediatric Hematology and Oncology, West China Second University Hospital, Sichuan University, Chengdu, China; 5 Department of Pharmacy, Hospital of Chengdu Office of People’s Government of Tibetan Autonomous Region, Chengdu, China; 6 Experimental Cancer Medicine, Clinical Research Center, Department of Laboratory Medicine, Karolinska Institute, Huddinge, 14186 Stockholm Sweden

## Abstract

In absence of direct comparison, we conducted an indirect-comparison meta-analysis to evaluate the efficacy and safety of thrombopoietin-receptor agonists(TPO-RAs) in treatment of pediatric persistent or chronic immune thrombocytopenia(ITP). PubMed, Embase, Cochrane Library, Clinical Trials.gov, China National Knowledge Infrastructure, and Chinese Biomedical Literature Database were searched from their earliest records to May 2017. Randomized controlled trials comparing the TPO-RAs with placebo in pediatric ITP were included. Outcomes included overall response rate(primary), durable response, overall or clinically significant bleeding, the proportion of patients receiving rescue medication, and safety. Five randomized placebo-controlled studies(*N* = 261) were analyzed. The overall response[*Risk Ratio(RR)* 0.57, 95% *confidence interval(CI)* 0.21–1.56], the incidence of adverse events (*RR* 0.96, 95%*CI* 0.66–1.39), durable response(*RR* 2.48, 95%*CI* 0.31–19.97), and the proportion of patients receiving rescue treatment(*RR* 0.73, 95%*CI* 0.20–2.73) were similar between eltrombopag and romiplostim group. Nevertheless, eltrombopag might have lower risk of overall bleeding(*RR* 0.43, 95%*CI* 0.23–0.80) and clinically significant bleeding(*RR* 0.33, 95%*CI* 0.12–0.89) than romiplostim. This meta-analysis suggests that eltrombopag might be similar to romiplostim in efficacy and safety, but seems to reduce the risk of bleeding compared to romiplostim. Furthermore, cost of the treatment, comorbidity of patients and drug compliance should also be considered in clinical decision making.

## Introduction

Immune thrombocytopenia (ITP) is an immune-mediated disease characterized by transient or persistent decrease in the platelet count and increased risk of bleeding^1^. The pediatric ITP incidence reported in recent population-based studies varied greatly in different countries: 1.91 per 10^5^ in Japan^2^, 2.83 per 10^5^ in France^3^, 4.2 per 10^5^ in the United Kingdom^4^ and 14.5 per 10^5^ in Korea^5^, respectively. Most children with ITP achieve remission spontaneously over time, however, 23.1%~47.3% of them develop a disease course of more than 6 months^6^.

For management of pediatric ITP, the first-line treatments include observation, corticosteroids, intravenous immunoglobulin (IVIg) and anti-D immunoglobulin^7–9^. If previous treatments fail, subsequent treatments may include splenectomy, rituximab, thrombopoietin receptor agonists (TPO-RAs), or more potent immunosuppression^7^. Eltrombopag (ELT), an oral non-peptide TPO-RA which could facilitate the proliferation and differentiation of megakaryocytes and increase platelet production^10^, has been approved by the United States Food and Drug Administration (FDA) for pediatric patients (≥1 year old) with chronic ITP who have “not achieved an appropriate response using other ITP medicines or splenectomy”^11^. Romiplostim (ROM), a subcutaneously administered peptide mimetic TOP-RA, was found effective in children with chronic ITP by randomized clinical trials and observational studies^12–19^. A systematic review including both adult and pediatric patients concluded that TPO-RAs substantially increased the rates of platelet response or durable response in children subgroup^20^.

Nevertheless, ROM binding to the extracellular TPO-receptor and ELT binding to a transmembrane site of the TPO-receptor have different mechanisms of action^21,22^. They also have different ways of administration, as ROM is given by subcutaneous injection, while ELT is given orally. Therefore, their efficacy and safety might be different. Unfortunately, there are no head-to-head randomized controlled trials (RCTs) comparing ROM with ELT in treatment of pediatric ITP. Hence indirect comparisons of the therapeutic outcomes across separate RCTs are recommended in the UK National Institute for Health and Clinical Excellence (NICE) methods guide^23^. The indirect comparison method proposed by Bucher preserves within-trial randomization by comparing treatment effects (*RR*) relative to a common comparator (placebo) from each trial, rather than comparing individual treatment arms from different trials^24^, and thus becomes a standard source of comparative evidence. An indirect comparison between ROM and ELT in treatment of adult patients with ITP was previously conducted^25^, but these conclusions may not be applicable for children due to different disease characteristics. This study aims to evaluate the efficacy and safety of ELT versus ROM for children with ITP using an indirect-comparison meta-analysis.

## Results

### Search result and characteristics of included studies

**Figure 1:**
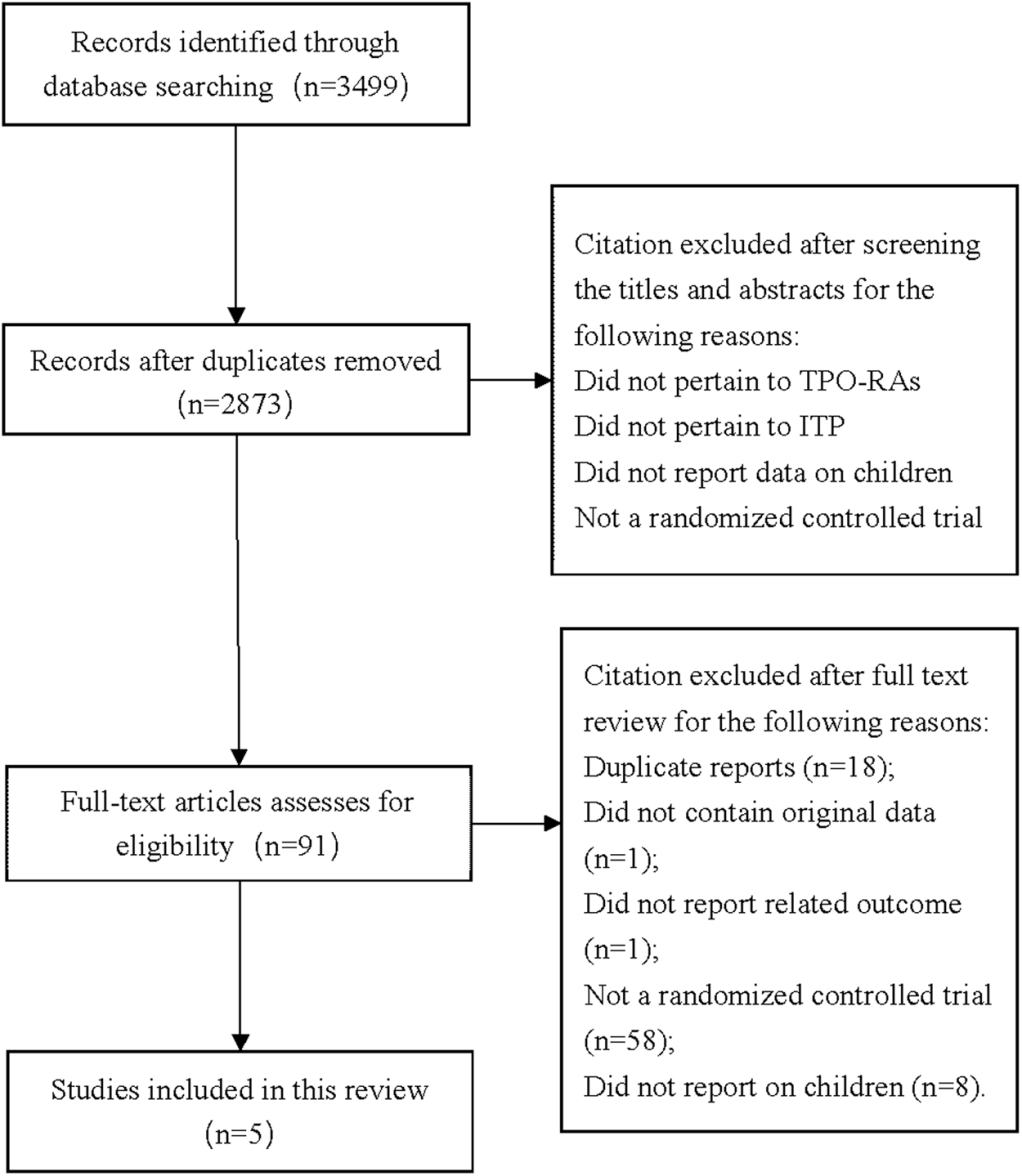
Flow diagram of study selection process for this systematic review.

A total of 3,499 citations were obtained from the literature search and Fig. 1 showed the selection process. Five randomized, placebo-controlled studies (261 participants)^18,19,26–28^ were included in this systematic review. Agreement between two reviewers for study selection was excellent (*K* = 0.89). As shown in Table 1, four multicenter studies were conducted in 16 countries or regions (USA, UK, Canada, Spain, France, the Netherlands, Argentina, Czech Republic, Germany, Hong Kong, Israel, Italy, Russia, Taiwan, Thailand and Australia)^18,19,27,28^ except one single-center study conducted in Egypt^26^.

**Table 1:** Characteristics of included studies.

Study ID	Study Design	Trial Registration Number	Population	Intervention vs Comparison	Outcome
Bussel 2015	Multicenter (22 centers in the USA, UK, Canada, Spain, France, and the Netherlands), double-blind, RCT.	NCT00908037	Patients aged 1–17years, with a diagnosis of ITP(duration of ≥6 months) in accordance with existing guidelines, had a platelet counts <30 × 10^9^/L.	Eltrombopag vs Placebo	①②③④⑤⑥⑦
Grainger 2015	Multicenter (38 centers in 12 countries: Argentina, Czech Republic, Germany, Hong Kong, Isreal, Italy, Russia, Spain, Taiwan, Thailand, UK, and USA), double-blind, RCT.	NCT01520909	Patients aged 1–17years, with a diagnosis of chronic ITP (duration of >12months) in accordance with the IWG guidelines, had a platelet counts <30 × 10^9^/L.	Eltrombopag vs Placebo	①②③④⑤⑥⑦
Bussel 2011	Multicenter (10 centers in the USA, Spain, and Australia), double-blind, RCT.	NCT00515203	Patients aged 1–17years, with a diagnosis with ITP(duration of ≥6 months) according to American Society of Hematology guidelines, had a platelet <30 × 10^9^/L.	Romiplostim vs Placebo	①③④⑤⑥⑦
Tarantino 2016	Multicenter (27 centers in the USA, Canada and Australia), double-blind, RCT.	NCT01444417	Patients aged 1–18 years with a diagnosis of primary ITP(duration of ≥6 months), had a platelet <0 × 10^9^/L.	Romiplostim vs Placebo	①②③④⑤⑥⑦
Elalfy 2011	Single-center (Egypt), single-blind, RCT.	NA	Patients aged 2.5–16 years with a diagnosis of ITP(duration of >12 months) according to American Society of Hematology guidelines, had a platelet <20 × 10^9^/L.	Romiplostim vs Placebo	①⑤⑥⑦

^①^Overall platelet response; ^②^Durable platelet response; ^③^Clinically significant bleeding; ^④^All bleeding events; ^⑤^Rescue medication; ^⑥^Adverse events; ^⑦^Serious adverse events; NA: not applicable.

All participants were aged 1–17 years old, with disease duration more than 6 months and baseline platelet count less than 30 × 10^9^/L. Two studies (159 participants) evaluated the efficacy and safety of ELT in comparison to placebo^27,28^ (Table 2). The initial dose of ELT was based on age and weight, and the following dose was adjusted according to individual platelet counts, with a target of 50–200 × 10^9^/L^27^. The initial dosage and adjustment were shown in Table 3. Three studies evaluated the efficacy and safety of ROM^18,19,26^. It was administrated at the initial dose of 1 μg/kg and was also adjusted according to platelet counts (Table 3). The double-blind phase of included studies ranged from 7 to 24 weeks.

**Table 2:** Characteristics of included patients.

Study ID	Participants(n): TPO-RA vs Control	Gender: Female/Male(n): TPO-RA vs Control	Age(years): TPO-RA vs Control	Duration of ITP(years): TPO-RA vs Control	Splenectomy status(yes/no)(n): TPO-RA vs Control	Baseline platelet count(10^9^/L): TPO-RA vs Control	Previous ITP medication(n): TPO-RA vs Control
Bussel 2015	45(ELT) vs 22(PLA)	27/18 vs 13/9	9(8–10) vs 10(8–12)	6–12 months: 8/45 vs 2/22 ≥12 months: 37/45 vs 20/22	5/40 vs 0/22	15.5 ± 8.0 vs 12.4 ± 8.8 PC ≤ 15 × 10^9^/L: 23/45 vs 11/22	≥2 agents: 38/45 vs 19/22
Grainger 2015	63(ELT) vs 29(PLA)	30/33 vs 14/15	9.4(8.2–10.5) vs 9.8(8.3–11.3)	3.4 ± 2.8 vs 4.4 ± 3.4	4/59 vs 0/29	PC ≤ 15 × 10^9^/L: 38/63 vs 19/29	≥1 agents: 60/63 vs 28/29 ≥2 agents: 46/63 vs 26/29
Bussel 2011	17(ROM) vs 5(PLA)	4/13 vs 2/3	9(1–17) vs 11(2–14)	2.4(0.8–14.0) vs 4.1(0.6–8.6)	6/11 vs 2/3	13(2–27) vs 9(8–29)	≥1 agents: 16/17 vs 5/5
Tarantino 2016	42(ROM) vs 20(PLA)	24/18 vs 11/9	10(6–14) vs 7.5(6.5–13.5)	1.9(1.0–4.2) vs 2.2(1.5–3.7)	1/41 vs 1/19	17.8(7.5–24.5) vs 17.7(9.8–24.1)	≥1 agents: 42/42 vs 20/20 ≥2 agents: 34/42 vs 14/20
Elalfy 2011	12(ROM) vs 6(PLA)	2/10 vs 3/3	9.5(2.5–16) vs 7(4–15)	2.3(1.2–7.0) vs 3.0(1.5–6.5)	0/12 vs 0/6	10.5(2–20) vs 10.5(6–20)	NA

ELT: Eltrombopag; ROM: Romiplostim; PLA: Placebo; PC: Platelet counts. NA: not applicable.

**Table 3:** The initial dose and dose adjustment of thrombopoietin-receptor agonists.

Study ID	Medication	TPO-RA Initial dose	Dose adjustment	Follow-up during double-blind phase
Bussel 2015	Eltrombopag	1–5years: 1.5 mg/kg/day (0·8 mg/kg once per day for east Asian patients). 6–11years: ≥27 kg: 50 mg/day or (25 mg/day for east Asian patients); <27 kg: 25 mg/day (12.5 mg/day for east Asian patient). 12–17years: 37.5 mg/day, orally.	Decreased dose by 12·5 mg/day if PC > 200 × 10^9^/L. Increased dose by 12·5 mg/day if PC < 50 × 10^9^/L, max 75 mg/d or 2 mg/kg/d. Interrupted treatment if PC > 400 × 10^9^/L.	7 weeks
Grainger 2015	Eltrombopag	1–5years: 1.2 mg/kg/day (0.8 mg/kg/day for east Asian patients). doses were adjusted based on platelet counts. 6–17yeas: ≥27 kg: 50 mg/day (25 mg/day for east Asian patients); <27 kg: 37·5 mg/day (25 mg/day for east Asian patients).	Doses were adjusted based on PC: Decreased dose if PC > 200 × 10^9^/L. Interrupted treatment if PC > 400 × 10^9^/L.	13 weeks
Bussel 2011	Romiplostim	1 μg/kg, once weekly.	Dose was adjusted to achieve target PC of 50 to 250 × 10^9^/L.	12 weeks
Tarantino 2016	Romiplostim	1 μg/kg, once weekly.	Dose was adjusted to achieve target paltelet counts of 50 to 200 × 10^9^/L.	24 weeks
Elalfy 2011	Romiplostim	1 μg/kg, once weekly.	Doses were escalated to 5 μg/kg and then tapered.	18 weeks

PC: Platelet counts.

### Risk of Bias assessment

**Figure 2:**
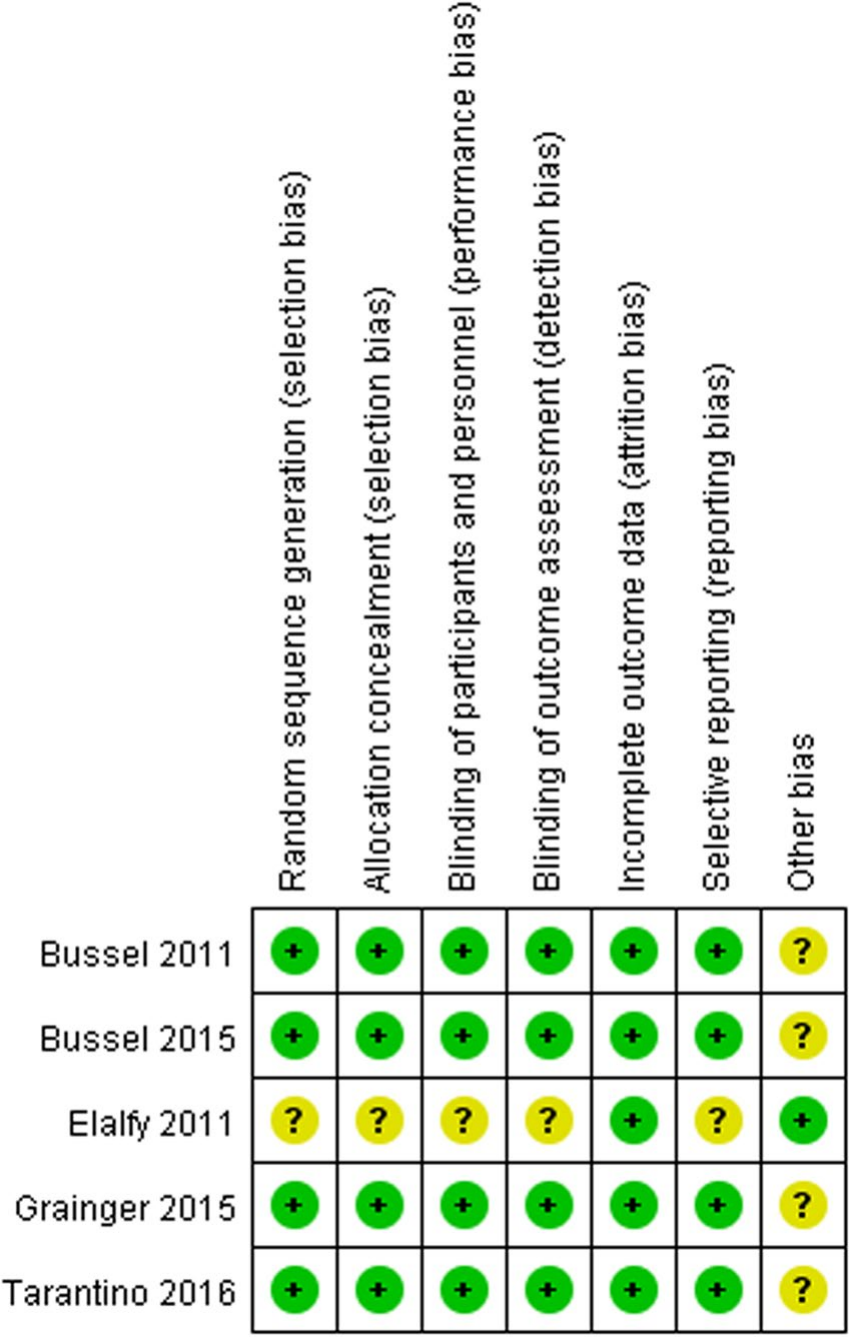
Risk of bias summary.

As shown in Fig. 2, four studies^18,19,27,28^ had low risk of selection bias for central randomization while the other one^26^ was unclear because the method of randomization and allocation concealment were not reported. Four studies^18,19,27,28^ had low risk of performance bias and detection bias, as both participants and study personnel were masked, however, this risk was not clear in one study^26^ for failing to report who was blinded. All studies^18,19,26–28^ had low risk of attrition bias, as there was no loss to follow-up or they dealt with the missing data properly (e.g. applying intention-to-treat analysis which could underestimate the efficacy of the interventions). Four studies^18,19,27,28^ had low risk of reporting bias since they were registered in *ClinicalTrials.gov* and had reported all predesigned outcome, however, the other study^26^ did neither mention registration information nor have an available protocol, so it was unclear whether all the pre-designed outcomes in this study had been reported. Four studies^18,19,27,28^ were supported by pharmaceutical industry, the bias caused by conflict of interest was unclear.

### Overall Platelet Response

**Figure 3:**
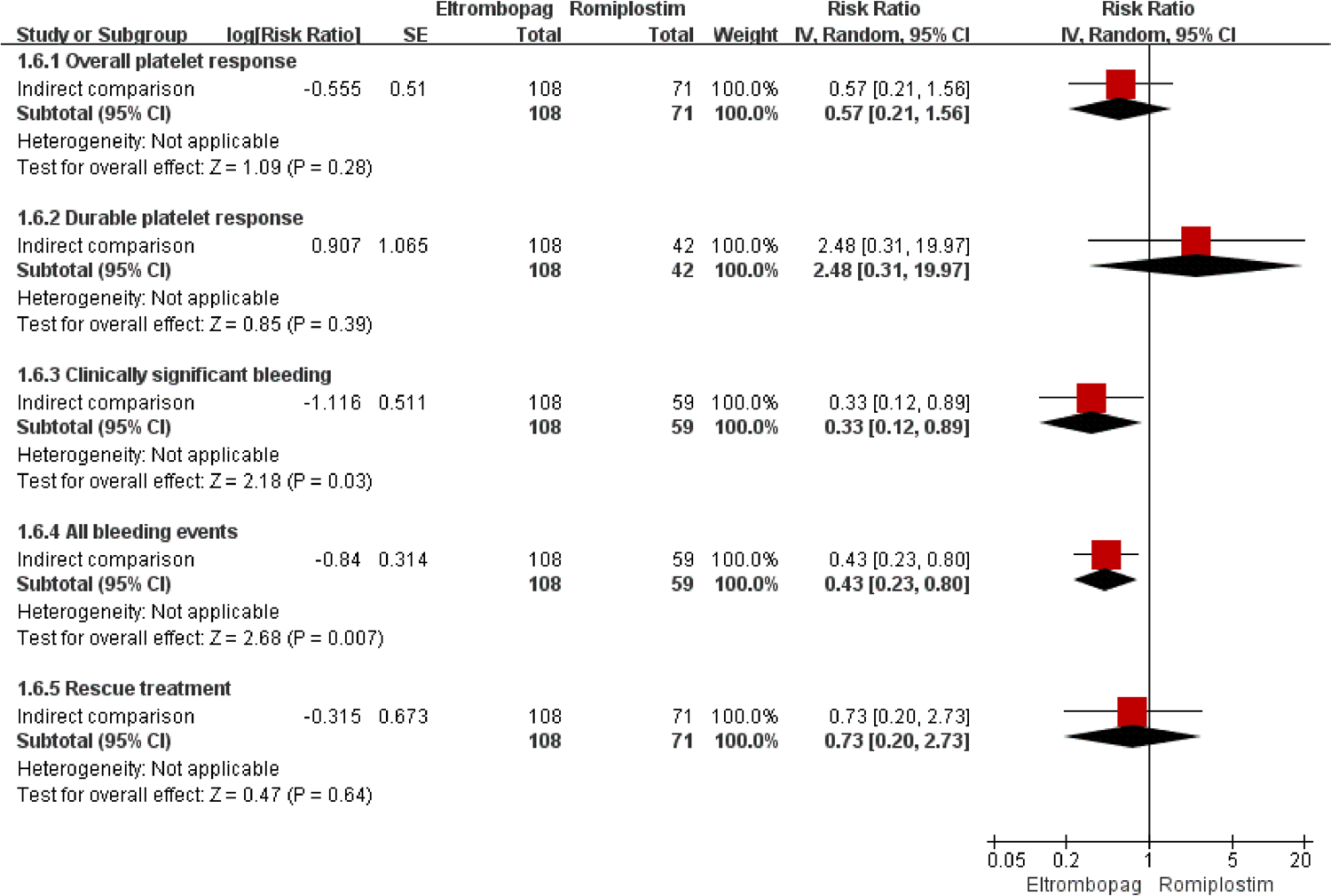
The efficacy results of indirect-comparison meta-analysis.

The overall platelet response was reported in all studies (two^18,19^ for ELT and three^26–28^ for ROM) including 261 participants. The heterogeneity was not statistically significant (*I*^2^ = 26%, *P* = 0.24 and *I*^2^ = 0%, *P* = 0.55, respectively). The pooled results with a fixed-effect model (Table 4) showed that proportion of participants achieving overall response was significantly higher in the TPO-RAs group than in the placebo group (*RR* = 2.64, 95%*CI*: 1.58–4.44, *P* < 0.05 for ELT and *RR* = 5.05, 95%*CI*: 2.21–11.53, *P* < 0.05 for ROM). The result of indirect comparison (Fig. 3) indicated that the overall response between ELT and ROM was not significantly different (*RR* = 0.57, 95%*CI*: 0.21–1.56, *P* > 0.05).

**Table 4:** The direct comparison meta-analysis results of outcomes.

Outcomes	TPO-RA vs PLA	n	N (TPO-RA vs PLA)	Heterogeneity	Model	*RR*	*95%CI*	*P*
Overall platelet response	ELT vs PLA	2	108 vs 51	*I*^2^ = 26%, *P* = 0.24	Fixed	2.64	[1.58, 4.44]	0.0002
	ROM vs PLA	3	71 vs 31	*I*^2^ = 0%, *P* = 0.55	Fixed	5.05	[2.21, 11.53]	0.0001
Durable platelet response	ELT vs PLA	2	108 vs 51	*I*^2^ = 0%, *P* = 0.83	Fixed	13.14	[2.67, 64.64]	0.002
	ROM vs PLA	1	42 vs 20	NA	NA	5.24	[1.36, 20.13]	0.02
Clinically significant bleeding	ELT vs PLA	2	108 vs 51	*I*^2^ = 0%, *P* = 0.39	Fixed	0.37	[0.15, 0.93]	0.04
	ROM vs PLA	2	59 vs 24	*I*^2^ = 0%, *P* = 0.94	Fixed	1.11	[0.78, 1.58]	0.57
All bleeding events	ELT vs PLA	2	108 vs 51	*I*^2^ = 63%, *P* = 0.10	Random	0.50	[0.29, 0.87]	0.01
	ROM vs PLA	2	59 vs 24	*I*^2^ = 0%, *P* = 0.42	Fixed	1.22	[0.89, 1.66]	0.21
Rescue treatment	ELT vs PLA	2	108 vs 51	*I*^2^ = 69%, *P* = 0.07	Random	0.46	[0.16, 1.34]	0.15
	ROM vs PLA	3	71 vs 31	*I*^2^ = 21%, *P* = 0.28	Fixed	0.70	[0.41, 1.20]	0.19
All adverse events	ELT vs PLA	2	107 vs 50	*I*^2^ = 73%, *P* = 0.06	Random	0.97	[0.72, 1.29]	0.82
	ROM vs PLA	2	29 vs 11	*I*^2^ = 0%, *P* = 1.00	Fixed	1.00	[0.69, 1.45]	1.00
Serious adverse events	ELT vs PLA	2	107 vs 50	*I*^2^ = 0%, *P* = 0.63	Fixed	0.70	[0.26, 1.86]	0.48
	ROM vs PLA	3	71 vs 30	*I*^2^ = 0%, *P* = 0.41	Fixed	3.28	[0.65, 16.60]	0.15
Headache	ELT vs PLA	2	107 vs 50	*I*^2^ = 0%, *P* = 0.70	Fixed	0.75	[0.41, 1.37]	0.35
	ROM vs PLA	3	71 vs 30	*I*^2^ = 0%, *P* = 0.92	Fixed	0.75	[0.46, 1.20]	0.23
Vomiting	ELT vs PLA	2	107 vs 50	*I*^2^ = 0%, *P* = 0.97	Fixed	0.31	[0.12, 0.82]	0.02
	ROM vs PLA	3	71 vs 30	*I*^2^ = 10%, *P* = 0.33	Fixed	0.85	[0.38, 1.87]	0.68
Upper respiratory tract infection	ELT vs PLA	2	107 vs 50	*I*^2^ = 0%, *P* = 0.87	Fixed	2.83	[0.88, 9.07]	0.08
	ROM vs PLA	2	59 vs 24	*I*^2^ = 0%, *P* = 0.45	Fixed	1.29	[0.59, 2.81]	0.52
Pyrexia	ELT vs PLA	2	107 vs 50	*I*^2^ = 0%, *P* = 0.60	Fixed	1.18	[0.39, 3.53]	0.77
	ROM vs PLA	2	59 vs 24	*I*^2^ = 0%, *P* = 0.81	Fixed	2.24	[0.63, 8.02]	0.21
Cough	ELT vs PLA	2	107 vs 50	*I*^2^ = 0%, *P* = 0.73	Fixed	5.24	[0.69, 39.64]	0.11
	ROM vs PLA	2	29 vs 11	*I*^2^ = 0%, *P* = 0.73	Fixed	0.36	[0.09, 1.47]	0.15
Epistaxis	ELT vs PLA	1	63 vs 29	NA	NA	0.61	[0.23, 1.61]	0.32
	ROM vs PLA	3	71 vs 30	*I*^2^ = 0%, *P* = 0.71	Fixed	0.95	[0.57, 1.59]	0.85
Oropharyngeal pain	ELT vs PLA	1	44 vs 21	NA	NA	2.39	[0.30, 19.16]	0.41
	ROM vs PLA	2	59 vs 24	*I*^2^ = 0%, *P* = 0.77	Fixed	4.28	[0.86, 21.26]	0.08
Abdominal pain	ELT vs PLA	2	107 vs 50	*I*^2^ = 45%, *P* = 0.18	Fixed	1.80	[0.46, 7.01]	0.40
	ROM vs PLA	1	17 vs 5	NA	NA	0.29	[0.02, 3.91]	0.35
Upper abdominal pain	ELT vs PLA	1	63 vs 29	NA	NA	0.35	[0.08, 1.44]	0.15
	ROM vs PLA	1	17 vs 5	NA	NA	2.33	[0.14, 38.97]	0.56
Diarrhoea	ELT vs PLA	1	44 vs 21	NA	NA	3.34	[0.44, 25.43]	0.24
	ROM vs PLA	1	42 vs 19	NA	NA	1.51	[0.47, 4.86]	0.49
Nausea	ELT vs PLA	1	44 vs 21	NA	NA	0.48	[0.17, 1.30]	0.15
	ROM vs PLA	1	42 vs 19	NA	NA	0.58	[0.25, 1.33]	0.20
Nasopharyngitis	ELT vs PLA	1	63 vs 29	NA	NA	2.53	[0.60, 10.70]	0.21
	ROM vs PLA	1	17 vs 5	NA	NA	1.67	[0.09, 30.06]	0.73

n: number of included studies; N: number of patients; ELT: Eltrombopag; ROM: Romiplostim; PLA: Placebo; *RR*: Risk Ratio; *CI*: confidence interval; NA: not applicable; Fixed: Fixed-effect model; Random: Random-effect model.

### Adverse events (AEs)

**Figure 4:**
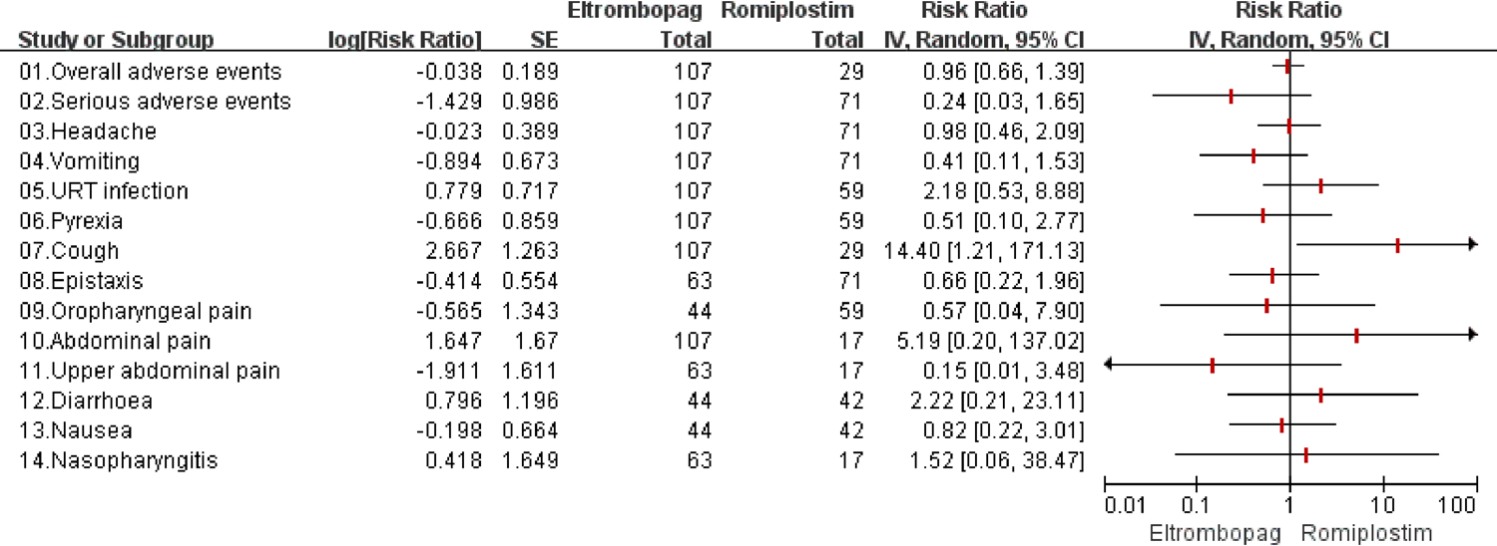
The safety results of indirect-comparison meta-analysis. URT: upper respiratory tract.

Four studies (197 participants)^19,26–28^ reported the overall incidence of any adverse events reported in participants receiving TPO-RAs or placebo. The pooled analysis (Table 4) showed that the incidence was not significantly different between two groups (*RR* = 0.97, 95%*CI*: 0.72–1.29, *P* > 0.05 for ELT and *RR* = 1.00, 95%*CI*: 0.69–1.45, *P* > 0.05 for ROM). And the result of indirect comparison (Fig. 4) also showed that the overall incidence of any AEs in ELT group was similar to that in ROM (*RR* = 0.96, 95%*CI*: 0.66–1.39, *P* > 0.05).

SAEs were reported in all studies^18,19,26–28^, and the results of both direct and indirect comparison (Table 4 and Fig. 3) indicated that the incidences of SAEs of ELT, ROM and placebo were not significantly different (ELT vs Placebo: *RR* = 0.70, 95%*CI*: 0.26–1.86, *P* > 0.05; ROM vs Placebo: *RR* = 3.28, 95%*CI*: 0.65–16.60, *P* > 0.05; ELT vs ROM: *RR* = 0.24, 95%*CI*: 0.03–1.65, *P* > 0.05; respectively).

The most common AEs in TPO-RAs and placebo were headache, vomiting, upper respiratory tract infection, pyrexia, cough, epistaxis, oropharyngeal pain, abdominal pain, and upper abdominal pain. However, the results of head-to-head comparison (Table 4) of the incidences demonstrated no significant difference between TPO-RAs and placebo, except that vomiting was less likely to occur in ELT than in placebo (*RR* = 0.31, 95%*CI*: 0.12-0.82, *P* < 0.05). According to the result of indirect comparison (Fig. 4), the incidences of these AEs in ELT were similar to that in ROM, except that cough was more frequent in ELT than in ROM (*RR* = 14.40, 95%*CI*: 1.21–171.13, *P* < 0.05).

### Durable Platelet Response

Three studies (221 participants)^18,27,28^ reported durable platelet response. The direct and indirect comparison analyses (Table 4 and Fig. 3) showed that the proportion of participants achieving durable platelet response in TPO-RAs was significantly higher than placebo (*RR* = 13.14, 95%*CI*: 2.76–64.64, *P* < 0.05 for ELT and *RR* = 5.24, 95%*CI*: 1.36–20.13, *P* < 0.05 for ROM), while there was no significant difference between ELT and ROM (*RR* = 2.48, 95%*CI*: 0.31–19.97, *P* > 0.05).

### Clinically significant bleeding

Four studies (242 participants)^18,19,27,28^ reported the incidence of clinically significant bleeding (grade 2~4). According to the direct comparison (Table 4), the incidence was significantly lower in participants receiving ELT than those receiving placebo (*RR* = 0.37, 95%*CI*: 0.15–0.93, *P* < 0.05), while the incidence was not significantly different between ROM and placebo (*RR = *1.11, 95%*CI*: 0.78–1.58, *P* > 0.05). As to indirect comparison (Fig. 3), the incidence was significantly lower in ELT group than in ROM group (*RR* = 0.33, 95%*CI*: 0.12–0.89, *P* < 0.05).

### All bleeding events

Four studies (242 participants)^18,19,27,28^ reported the incidence of all bleeding events (grade 1–4). The pooled results (Table 4) demonstrated that the incidence was significantly lower in ELT group compared with placebo group (*RR* = 0.50, 95%*CI*: 0.29–0.87, *P* < 0.05), while the incidence was not statistically different between ROM and placebo (*RR* = 1.22, 95%*CI*: 0.89–1.66, *P* > 0.05). According to the indirect comparison (Fig. 3), the incidence was significantly lower in ELT group than in ROM group (*RR* = 0.43, 95%*CI*: 0.23–0.80, *P* < 0.05).

### Rescue treatment

All studies (261 participants)^18,19,26–28^ reported the proportion of participants receiving rescue treatment in TPO-RAs or placebo group. The results of both direct and indirect comparison (Table 4 and Fig. 3) indicated that the proportion was not significantly different between ELT, ROM and placebo. (ELT vs Placebo: *RR* = 0.46, 95%*CI*: 0.16–1.34, *P* > 0.05; ROM vs Placebo: *RR* = 0.70, 95%*CI*: 0.41–1.20, *P* > 0.05; ELT vs ROM: *RR* = 0.73, 95%*CI*: 0.20–2.73, *P* > 0.05; respectively).

## Discussion

This is the first systematic review incorporating an indirect-comparison meta-analysis summarizing the evidence of efficacy and safety of TPO-RAs in children with persistent or chronic ITP. Our study suggests that the use of TPO-RAs may improve the durable and overall platelet response compared with placebo, while ELT resembles ROM in efficacy. It’s shown that ELT might be superior to ROM or placebo in reducing bleeding risk (including clinically significant bleeding). The incidences of AEs (including SAEs) of ELT, ROM and placebo were similar. Neither ELT nor ROM could reduce the need for rescue treatment.

A systematic review on the efficacy and safety of TPO-RAs in adults and children concluded that TPO-RAs were effective and safe second-line options for primary ITP patients^20^. According to its subgroup analysis for children, TPO-RAs could significantly improve platelet response (*RR* = 2.49, 95%*CI*: 1.46–4.23, *P* < 0.05) and durable response (*RR* = 7.64, 95%*CI*: 2.73–21.36, *P* < 0.05), without increasing the incidence of AEs (including SAEs) compared with placebo or standard care. Those findings are mostly consistent with the results of this review on children. However, our research indicated that ELT might reduce the risk of bleeding compared to ROM or placebo, and the TPO-RAs did not reduce the need for rescue treatment, which are inconsistent with previous study results. These differences might be caused by the different ways in pooling data: the previous study pooled the results from studies on ELT and ROM together, while our study conducted analysis on the two drugs separately.

An indirect comparison conducted in adult ITP patients demonstrated that ROM significantly improved overall platelet response compared to ELT, while the durable platelet response of the two TPO-RAs was similar^25,29^. Our research drew a different conclusion that ELT and ROM were similar in overall or durable platelet response for children with persistent or chronic ITP, probably due to population difference.

In addition, a multicenter retrospective study including 87 pediatric ITP patients (36 in ELT and 51 in ROM, respectively)^17^ found that the durable platelet response (*RR* = 1.00, 95%*CI*: 0.76–1.30, *P* > 0.05), overall platelet response (*RR* = 0.93, 95%*CI*: 0.77–1.13, *P* > 0.05), the incidence of AEs (*RR* = 2.83, 95%*CI*: 0.27–30.07, *P* > 0.05), and the proportion of patients receiving rescue treatment (*RR* = 0.89, 95%*CI*: 0.46–1.72, *P* > 0.05) were not significantly different between ELT and ROM, which are also consistent with the results of our study. Similar findings were also reported in other small sample observational studies^30,31^.

Nonetheless, an updated systematic review and meta-analysis of RCTs suggested that TPO-RAs were associated with higher risk of thromboembolic events compared with placebo or standard care^32^. And another meta-analysis of three large, population-based observational studies concluded that the risk of arterial and venous thromboembolism should be considered when evaluating the risk of thromboembolism attributed to ITP treatments (e.g. TPO-RAs)^33^. A disproportionality analysis in the World Health Organization global individual case safety report (ICSR) database (VigiBase) suggested the presence of a signal for an increased risk of thrombosis with ELT compared to ROM (adjusted reporting odds ratio = 1.72, 95%*CI* 1.47–2.02, *P* < 0.05)^34^. Although these AEs were not reported in the RCTs due to small sample size, physicians should be cautious when administering TPO-RAs to patients with higher risk of thromboembolism, especially for ELT.

In spite of increasing pharmacoeconomic studies revealing the advantage of ELT in cost-effectiveness for adult patients with ITP^35–37^, further studies are still needed to compare the benefits and cost of different TPO-RAs for pediatric persistent or chronic ITP to facilitate better clinical decision making.

There are several limitations in this study. As we only included RCTs in this review, the results may not have good generalizability for strict inclusion criteria and small sample size in those studies. These studies were also not sensitive enough to find rare AEs related to the drugs, as the sample size was relatively small. On the other hand, the results of indirect comparisons between ELT and ROM should be interpreted with caution because of low power of test and heterogeneity caused by different study designs, patient populations, and outcome definitions.

In summary, ELT and ROM might lead to similar overall and durable platelet response and incidence of adverse events in children with persistent or chronic ITP, but patients receiving ROM might have lower risk of bleeding compared to those receiving ROM. Cost of treatments, comorbidity and drug compliance should also be considered when making decisions in the management of pediatric persistent or chronic ITP with thrombopoietin-receptor agonists.

## Methods

We followed the standard set by Preferred Reporting Items for Systematic reviews and Meta-Analyses (PRISMA) in this systematic review (Table 1). The study was registered in PROSPERO International Prospective Register of Systematic Review (PROSPERO 2017: CRD42017068657).

### Searching

Pubmed, Embase, and Cochrane Central Register of Controlled Trials (CENTRAL) published in Cochran Library were searched using the search strategies detailed in Table S2, from their earliest records to May 2017. Clinical Trials.gov was searched using the terms “immune thrombocytopenia”, “children”, “eltrombopag”, and “romiplostim”. The China National Knowledge Infrastructure(CNKI) and Chinese Biomedical Literature Database(CBM) were also searched in Chinese.

### Eligibility Criteria

All included studies met the following criteria: (1) randomized controlled studies; (2) participants were children <18 years with ITP lasting for 6 months or longer; (3) the intervention was ELT or ROM irrespective of dosage and schedule; (4) the comparison was placebo; (5) studies included at least one of the following outcomes: overall platelet response (primary outcome), defined as achieving at least once platelet response (50 × 10^9^/L or more) during treatment; incidence of overall and serious adverse events (SAEs); durable platelet response, defined as achieving platelet counts of 50 × 10^9^/L or more for 4 or more weeks; incidence of clinically significant bleeding (WHO Grade 2~4); all bleeding events; and the proportion of patients who received rescue treatment[e.g. receiving any unscheduled or new treatment (including new drugs, increase the dose of a concomitant drug from baseline, platelet transfusion or splenectomy) for immediate risk or treatment failure]; (6) publication written in English or Chinese. We excluded the patients with Evans syndrome or secondary ITP and the studies including both children and adults when children data could not be extracted separately.

### Study Selection and Data Extraction

Two authors independently screened the titles and abstracts of all studies identified by the search strategies, and assessed the studies using predetermined inclusion criteria. The full texts of all potentially relevant articles were retrieved for detailed review. We resolved any disagreements by discussion until consensus was achieved. We used a pre-designed data collection form to extract data from each eligible study. The following data were extracted: (1) authors; (2) year of publication; (3) country or region where the study conducted; (4) study design and use of control; (5) number of participants randomized into each group; (6) gender, age, and disease duration of participants; (7) baseline platelet count, previous ITP medication, and splenectomy status; (8) dose and schedule of TPO-receptor agonists; (9) outcomes of each study and their definitions; (10) numerical data for assessment of included outcomes; (11) sources of funding.

### Risk of Bias Assessment

Two authors independently assessed the risk of bias of each included study using the checklist developed by Cochrane Collaboration^38^. The items included random sequence generation, allocation concealment, blinding, incomplete outcome data, selective outcome reporting, and other bias. We categorized the judgments as low, high or unclear risk of bias and created plots of risk of bias assessment in Review Manager Software (RevMan 5.3).

### Statistical Synthesis

We calculated a kappa statistic for measuring the agreement level between two authors making decisions on study selection. The value of kappa (*K*) between 0.40 and 0.59 was considered as fair agreement, between 0.60 and 0.74 as good and 0.75 or more as excellent.

If more than one study reported the same outcome, the pairwise meta-analysis was conducted to calculate the pooled estimate of the risk ratio (*RR*) of different TPO-RAs versus placebo by RevMan 5.3. Statistical heterogeneity among studies was examined by the Chi-square test and quantified by the *I*^2^ statistic^39^. We used a fixed-effect model to synthesize data when heterogeneity was not significant (*P* > 0.1 and I^2^ < 50%). When heterogeneity was significant (*P* ≤ 0.1 and I^2^ ≥ 50%) and could not be explained by subgroup analyses or in terms of clinical or methodological features of the trials, the random-effect model was used. If both the ELT-placebo and ROM-placebo trials reported the same outcome, the relative treatment effect (*RR*) for ELT versus ROM was estimated using indirect procedure of Stata12.0 software^40^, with the formula as follow:

$$R{R}_{\mathrm{ELT}/\mathrm{ROM}}=R{R}_{\mathrm{ELT}/\mathrm{Placebo}}/R{R}_{{\rm{ROM}}/{\rm{Placebo}}},$$

$${\rm{Variance}}(\mathrm{log}\,R{R}_{\mathrm{ELT}/\mathrm{ROM}})={\rm{Variance}}(\mathrm{log}\,R{R}_{\mathrm{ELT}/\mathrm{Placebo}})+{\rm{Variance}}(\mathrm{log}\,R{R}_{{\rm{ROM}}/{\rm{Placebo}}})$$

The subgroup analysis was also conducted according to the different types of adverse events.
